# *Burkholderia* *pseudomallei* Type G in Western Hemisphere

**DOI:** 10.3201/eid2004.130960

**Published:** 2014-04

**Authors:** Jay E. Gee, Christopher J. Allender, Apichai Tuanyok, Mindy G. Elrod, Alex R. Hoffmaster

**Affiliations:** Centers for Disease Control and Prevention, Atlanta, Georgia, USA (J.E. Gee, M.G. Elrod, A.R. Hoffmaster);; Northern Arizona University, Flagstaff, Arizona, USA (C.J. Allender, A. Tuanyok)

**Keywords:** melioidosis, Western Hemisphere, internal transcribed spacer, molecular typing, *Burkholderia* *pseudomallei*, type G, bacteria

## Abstract

*Burkholderia pseudomallei* isolates from the Western Hemisphere are difficult to differentiate from those from regions in which melioidosis is traditionally endemic. We used internal transcribed spacer typing to determine that *B. pseudomallei* isolates from the Western Hemisphere are consistently type G. Knowledge of this relationship might be useful for epidemiologic investigations.

*Burkholderia pseudomallei* is the causative agent of the disease melioidosis. Melioidosis is considered endemic to Southeast Asia and northern Australia. However, sporadic cases do occur elsewhere in the world, especially in tropical areas (*1*).

The predominant method of molecular subtyping of *B. pseudomallei* is multilocus sequence typing (MLST), which is based on a comparison of the alleles of 7 housekeeping genes to generate a sequence type (ST) (*2*). These data can then be analyzed by using tools such as eBURST, which is used to infer phylogenetic patterns (*3*). 

As of May 30, 2013, a total of 3,028 *B. pseudomallei* isolates were listed in the MLST database (www.mlst.net); these isolates are predominantly from Southeast Asia (1,036 isolates) and Australia (1,776). Some entries are from other Pacific areas (e.g., New Caledonia [9 isolates] and Hong Kong [39]) and other parts of the world (e.g., Africa [8], Europe [15], and the Western Hemisphere [30]) or of unknown origin (32).

For a population study, Pearson et al. analyzed the STs in the *B. pseudomallei* MLST database along with other data, such as single-nucleotide polymorphisms from whole-genome sequencing data (*4*). Their study indicated some population structures associated with geographic origin, in particular, clades associated with isolates from Southeast Asia or northern Australia. The data were used to support a hypothesis that *B. pseudomallei* originated on the Australian continent, spread to Southeast Asia, and from there spread throughout the world (*4*).

For an isolate of unknown origin, however, MLST alone may not provide information about geographic origin. For example, isolates from the Western Hemisphere have yielded STs unique to that hemisphere, but when these isolates were analyzed by eBURST or other methods, no distinct clade was found. In the clusters in which these STs appear, STs are intermixed with those from regions in which melioidosis is traditionally endemic, such as Southeast Asia (*2*,*4*). Therefore, a method for determining whether an isolate of unknown origin is from the Western Hemisphere is desirable.

Recently, Ligouri et al. developed a typing scheme by measuring the length polymorphisms in the 16S–23S internal transcribed spacer (ITS) of *Burkholderia* spp. (*5*). The typing scheme consists of 10 types: A, B, C, D, E, F, G, CE, GE, and GC. Ligouri et al. found that some types are unique to a given *Burkholderia* species (i.e., A = *B. thailandensis*, B = *B. humptydooensis*, D = *B. oklahomensis*, and *F* = *B. cepacia*). They also determined that type C could be found in *B. mallei* and in *B. pseudomallei*. The remaining 5 types were exclusive to *B. pseudomallei* (*5*). Ligouri et al. determined that types C, E, GE, and CE were the predominant types for isolates from northern Australia and Southeast Asia and that type G was rare in Australia (4 isolates) and Southeast Asia (3). They noted, on the basis of a limited number of strains from these regions, that type G was overrepresented in isolates from other parts of the world: Madagascar (1 isolate), Ecuador (2), Puerto Rico (2), Venezuela (1), and Kenya (1). They hypothesized that a genetic bottleneck occurred during the dispersal of type G to regions outside of Southeast Asia and Australia (*5*).

ITS typing of *B. pseudomallei* might be a powerful tool for linking cases of melioidosis to regions outside of those in which melioidosis in highly endemic, such as Southeast Asia and northern Australia. To further investigate this trend, we assessed the ITS types of *B. pseudomallei* from the Western Hemisphere.

## The Study

All tested Western Hemisphere isolates from our collection were ITS type G (Table). In addition to those isolates tested, we performed in silico analysis of whole-genome sequencing data from other *B. pseudomallei* isolates in our collection or publicly available data with origins in the Western Hemisphere and found them to also be ITS type G (Table). As expected, eBURST analysis of the STs from the type G strains that originated in the Western Hemisphere did not yield a discrete clade. These STs are interspersed with other STs that are predominantly from Southeast Asia (Figure). Our results support the findings of Ligouri et al. (*5*). The predominance of type G in isolates in our panel of isolates from the Western Hemisphere is consistent with a hypothesis that these isolates are derived from a bottleneck that occurred during dispersal to the rest of the world from Southeast Asia.

**Table:** Analysis results for *Burkholderia pseudomallei* isolates from Western Hemisphere*

Identification no.	ITS type	YLF gene	MLST	Source
Swiss2010	G	+	92	Switzerland ex Martinique 2010
PR1998	G†	+†	92	Puerto Rico 1998 (GenBank accession no. pending)
4900CF	G†	+†	92	Brazil: cystic fibrosis patient 2007 (GenBank accession no. ARZE00000000)
CA2009	G	+	95	USA (California) ex Mexico 2009
FL2009	G	+	297	USA (Florida) ex Puerto Rico 2009
FL2012	G	+	297	USA (Florida) ex Trinidad 2012
PR2012	G	+	297	Puerto Rico 2012
MX2013	G	ND	297	Mexico 2013
PB08298010	G†	+	426	USA (Arizona) locally acquired 2008 (GenBank accession no. ARZO00000000)
CA2012d	G	+	436	USA (California) ex Guatemala 2012
2002734728	G	+	518	USA (California) Iguana 2007
PB 1007001	G	+	518	USA (Arizona) ex Costa Rica 2010
CA2013a	G	ND	518	USA (California) Iguana 2013
NY2010	G	+	698	USA (New York) ex Aruba 2010
724644	G	+	698	USA (Massachusetts) ex Aruba 2010
BCC215	G†	+†	ND‡	Brazil (Ceara) 2003 (GenBank accession no. ABBR00000000)

*ITS, internal transcribed spacer; YLF, *Yersinia*-like fimbrial; MLST, multilocus sequence type; ex, diagnosis made in first location but infection acquired in second location; ND, not determined. †Determined by analysis of whole-genome sequence. ‡Allele for gltB not available.

**Figure 1:**
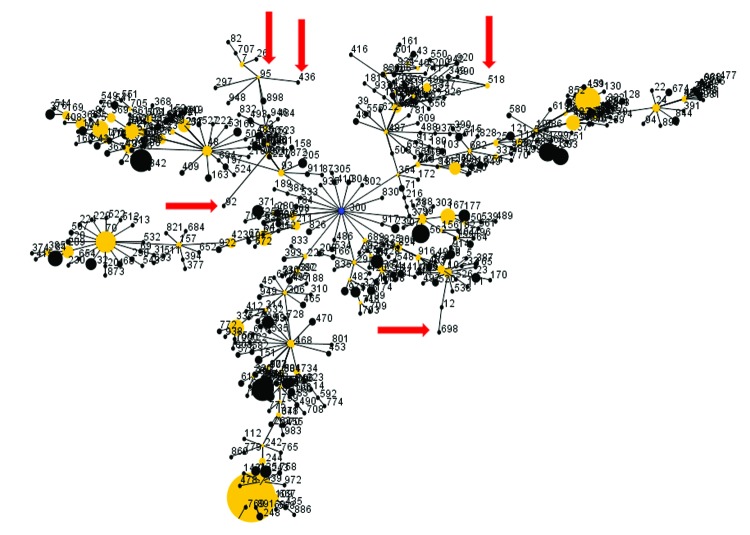
Diagram of eBURST (*3*) analysis of multilocus sequence types (STs) of *Burkholderia pseudomallei* isolates; default settings were used. Dots represent sequence types. Blue dot represents the calculated primary founder, yellow dots represent calculated subgroup founders, and black dots represent the remaining STs. The sizes of dots are proportional to the number of isolates in the database representing a given ST. A single line between sequence types indicates that they are single-locus variants of each other. Red arrows indicate positions of STs associated with isolates from the Western Hemisphere and that are type G according to internal transcribed spacer typing.

Other supporting evidence is provided by testing for the *Yersinia*-like fimbrial (YLF) gene by YLF PCR. The presence of the YLF gene is associated with *B. pseudomallei* isolates from Southeast Asia (*6*). For those strains tested, all were positive for YLF or the YLF gene was present according to in silico analysis of whole-genome sequencing data when available (Table).

## Conclusions

In melioidosis cases for which the origin is unclear, such as for patients with unknown exposure histories or histories of travel to multiple regions to which melioidosis is endemic, ITS typing along with other molecular epidemiologic tools might be useful for assessing the *B. pseudomallei* origins. More insight into the relationship of Western Hemisphere isolates to isolates from regions to which melioidosis is highly endemic might come from whole-genome sequencing. As more isolates are analyzed, such studies might enable higher confidence in the geographic origin of isolates.
